# Enhancing fall risk awareness among high fall-risk inpatients: a best practice implementation project

**DOI:** 10.1186/s12912-026-04891-7

**Published:** 2026-06-18

**Authors:** Caifang Chen, Sirong Wu, Yang Zhou, Yu Dai, Yueping Zheng, Liaofang Wu, Hua Chen, Jinping Zhou, Meiling Hu, Jingcan Xu

**Affiliations:** 1 Teaching and Research Section of Clinical Nursing, Xiangya Hospital of Central South University, No. 87 Xiangya Road, Kaifu District, Changsha, Hunan 410008 China; 2 Day Surgery Center, Xiangya Hospital of Central South University, Changsha, Hunan China; 3 Xiangya School of Nursing, Central South University, Changsha, Hunan China

**Keywords:** Inpatients, Falls, Risk awareness, Evidence-based nursing

## Abstract

**Introduction:**

Falls are one of the most common adverse events among inpatients. Patients’ awareness of fall risk can influence patient’ behaviors and potentially reduce fall incidence. Although the importance of risk awareness in falls prevention has been increasingly recognized, how to effectively enhance awareness among high fall-risk inpatients and its consequent impact on fall rates remains to be further validated.

**Objective:**

To develop and implement an evidence-based practice protocol to enhance fall risk awareness among high fall-risk inpatients and evaluate its effectiveness, and to clarify its unique value compared with existing fall prevention approaches.

**Methods:**

This study constituted a nonrandomized, pre–post quasi-experimental evidence implementation project following the JBI PACES (Practical Application to Clinical Evidence System). The project was conducted in seven inpatient wards with the highest fall incidence rates over the preceding three years at a tertiary hospital in Changsha, China. A total of 420 high- risk inpatients (210 at baseline, 210 post-implementation) and 70 nurses from these wards were enrolled. Through evidence synthesis, 22 best practice recommendations were initially identified. Following FAME (feasibility, appropriateness, meaningfulness, and effectiveness) evaluation, 18 recommendations were retained, from which 20 audit criteria were developed. A baseline audit was conducted among 210 inpatients (June–July 2024) and the 70 nurses to evaluate compliance with these criteria. Barriers to best practice implementation were systematically identified through baseline audit findings using the JBI Getting Research into Practice (GRiP) framework, and a concise targeted implementation strategy was developed and applied for three months. This program employed multifaceted strategies across five dimensions: organizational support, system optimization, staff training, patient education, and electronic medical record (EMR)-integrated warning systems. A follow-up audit was then conducted (October–December 2024). Outcome measures, including audit criteria compliance rates, fall and injury incidence, patients’ fall risk perception scores, patients’ knowledge, attitude, and behavior regarding fall prevention and nursing staff’s knowledge and attitudes, were compared pre- and post-implementation.

**Results:**

Following EBP implementation, 16 of the 20 audit criteria demonstrated significant improvement (p < 0.01). Notably, nine criteria achieved 100% compliance, and seven criteria increased from 0% to 100%. The incidence of falls decreased from 0.48% (1/210) to 0% (0/210), and the injury rate decreased correspondingly from 0.48% to 0%. Patients’ Fall Risk Perception Scores increased significantly post-EBP: at admission, the mean score increased from 15.5 (SD 12.93) to 20.21 (SD 13.52); at discharge, from Mean score 16.8 (SD 12.69) to Mean score 28.15 (SD 19.76). Patients’ scores across the three dimensions of fall prevention knowledge, attitudes, and behaviors were significantly higher post-EBP (p < 0.01). Nursing staff demonstrated significantly improved knowledge on five fall risk awareness-related items (p < 0.01), and their attitude scores were significantly higher post-intervention (P < 0.01).

**Conclusions:**

This evidence-based practice protocol effectively enhanced fall risk awareness among high fall-risk inpatients, improved their knowledge, attitudes, and behaviors regarding fall prevention, enhanced nursing staff’s knowledge and attitudes, and improved compliance with audit criteria. These findings provide a scientific basis for integrating patient-centered risk communication into routine fall prevention programs for fall high-risk inpatient populations.

**Trial registration:**

This evidence implementation project was retrospectively registered with the Fudan University Centre for Evidence-based Nursing—a JBI Centre of Excellence. This was not a randomized controlled trial.

**Clinical trial number:**

ER20251072.

**Registration date:**

9 January 2025.

**Supplementary Information:**

The online version contains supplementary material available at https://doi.org/10.1186/s12912-026-04891-7.

## Introduction

This evidence implementation project was retrospectively registered with the Fudan University Centre for Evidence-based Nursing—a JBI Centre of Excellence. This was not a randomized controlled trial.

Falls represent one of the most prevalent adverse events in hospitalized patients, resulting in physical injuries, prolonged hospitalization, psychological distress, and substantial healthcare costs [1]. Studies have reported that the incidence of falls among inpatients ranges from 1.36 to 11.5 per 1,000 patient-days, with approximately 26%-42% of these falls resulting in injuries [2, 3]. In high-risk populations—such as older adults and patients with cognitive impairment—falls are more likely to result in severe injuries such as fractures, head trauma, or prolonged functional impairment. A multicenter study by Xu et al. [4] analyzing administrative data from over 10,000 hospitalized adults identified multiple independent risk factors for serious fall injuries, including advanced age, cognitive impairment, and use of sedatives or anti-hypertensives. Their findings underscore that falls in vulnerable patients not only occur frequently but also carry disproportionately high risks of severe outcomes, highlighting the urgent need for targeted, patient-centered prevention strategies tailored to individual risk profiles. Fall-related injuries not only compromise patient safety and quality of life but also impose significant economic burdens on healthcare systems, with estimated annual costs exceeding billions of dollars globally [5]. Despite the implementation of various preventive measures in clinical settings—such as environmental modifications, patient education, and nursing surveillance, fall rates remain unacceptably high, suggesting that existing interventions may not adequately address the multifaceted nature of fall risk or sufficiently engage high-risk patients in their own care [6].

Given this persistent challenge, growing attention has been directed toward patients’ subjective awareness of their own fall risk. Patients’ awareness of their fall risk directly influences their engagement in preventive behaviors [7]. Waszynski et al. [8] further demonstrated that operationalizing fall risk awareness through patient-family co-designed bedside visual tools achieved a 43% reduction in falls, suggesting such awareness functions as a catalyst for measurable intervention rather than merely an educational endpoint. Understanding these perspectives is essential for informing clinical fall prevention policies [9]. Research indicates that individuals with accurate risk awareness are more likely to adopt protective behaviors, such as requesting assistance when ambulating or using handrails, thereby reducing their actual fall risk [10]. Conversely, insufficient insight regarding personal fall risk correlates with increased incidence of falls and reduced adherence to prevention strategies [11, 12]. For instance, overconfident patients may underestimate their vulnerability and reject necessary assistance, while excessive fear of falling may lead to activity restriction and functional decline [13]. Therefore, enhancing patients’ fall risk awareness represents a critical yet underexplored avenue for improving fall prevention outcomes, particularly among those identified as high-risk.

However, few studies have translated fall risk awareness evidence into a structured clinical protocol, and its incremental value over traditional fall prevention remains unclear.Fall risk awareness varies significantly across diverse populations, with existing research primarily focusing on older adults with chronic conditions, such as those with cardiovascular disease [14, 15]. In hospitalized settings, this awareness is shaped by multiple determinants, encompassing individual characteristics (e.g., age, education level, and cognitive status) alongside clinical factors including disease type, medication use, and treatment regimen [16]. Recently, our team conducted a comprehensive evidence summary on enhancing fall risk awareness among hospitalized patients, synthesizing 22 best practice recommendations across five domains: organizational management, risk awareness assessment, individualized intervention, training and education, and informatization and visualization [17]. This evidence summary provided the foundational framework for the present study. Nevertheless, while that evidence summary identified what evidence exists, how to translate these components into a structured, implementable clinical protocol and evaluate its effectiveness in real-world settings remained unexplored. The translation of existing evidence into clinical practice encounters substantial barriers, including organizational management constraints, appropriate application of assessment tools, lack of standardized educational interventions, and the individualization of strategies to diverse patient needs [18]. Therefore, building upon the previously synthesized evidence, this study aimed to develop a multi-dimensional intervention protocol to enhance fall risk awareness among high fall-risk inpatients, and evaluate its effectiveness in clinical practice, thereby providing a scientific foundation for fall prevention in this vulnerable population.

## Materials and methods

The study was approved by the Medical Ethics Committee of Xiangya Hospital, Central South University, (No.202310212). Written informed consent was obtained from all participants. Prior to enrollment, research staff explained the study purpose, procedures, and rights of withdrawal. This study constituted a nonrandomized, pre–post quasi-experimental evidence implementation project following the JBI Practical Application to Clinical Evidence (JBI PACES) and Getting Research into Practice (GRiP) frameworks. It is reported in accordance with the SQUIRE 2.0 (Standards for QUality Improvement Reporting Excellence) guidelines for quality improvement reporting [19]. The project was conducted in seven inpatient wards with the highest fall incidence rates over the preceding three years at a tertiary hospital in Changsha City, Hunan Province, China. These units included Neurology (comprising Geriatric Neurology and General Neurology), General Surgery, Endocrinology (comprising Geriatric Endocrinology and General Endocrinology), Geriatric Cardiology, and Gynecology. This study included 210 patients with high-risk fall assessment scores on the Morse Fall Scale between June 1 to July 31, 2024 as the baseline review subjects, Another 210 high-risk fall patients hospitalized between October 1 to December 31,2024 were selected as the post-evidence-based practice study subjects. Patient inclusion criteria were as follows: (1) Hospitalized patients aged ≥ 18 years; (2) High-risk results on the fall risk assessment scale(score ≥ 45);(3) able to communicate verbally based on clinical judgement, with no diagnosis of severe dementia, aphasia, or other conditions impairing communication documented in medical records; (4) Voluntary participation in the study with signed informed consent. Exclusion criteria: Patients who met one or more of the following criteria were excluded: (1) Individuals with intellectual or mental disorders; (2) hearing or communication impairments that precluded questionnaire completion even with assistance. Additionally, this study included 70 nurses from the seven units. Nurse inclusion criteria were as follows: (1) Registered nurses who had worked in the seven units for ≥ 1 year. Exclusion criteria were: (1)in the evidence review team, (2) on maternity or sick leave for ≥ 7 days during the study period, (3) off-site for training or study for ≥ 7 days during the study period, and (4) interns, trainees, or student nurses. This study utilized the JBI PACES and GRiP tools to effectively translate research evidence into clinical practice application. The tool application was divided into three stages: (1) Establishing the project team, developing audit criteria based on the best evidence, and conducting a baseline audit; (2) Based on the baseline audit results, the project team designed various strategies to address non-compliance issues and barriers identified in the baseline audit; (3) Conducting a follow-up audit to evaluate the effectiveness of the interventions implemented to improve practice and identify practice issues requiring attention in future research.

### Phase 1: Establishment of an evidence-based practice team and conducting a baseline review

#### Establishment of an evidence-based practice team

The project was led by the deputy director of the nursing department, who provided coordination and support. The District Nurse Manager acted as the deputy team leader, responsible for project coordination and promotion. An Information Technology Specialist was responsible for integrating tools to enhance fall risk awareness into the Electronic Medical Record (EMR) system. A Pharmacist was responsible for coordinating improvements related to medications. A Medical Specialist was included to provide clinical expertise and ensure alignment with the best medical practices. Seven head nurses and seven nurses of participating wards acted as project members, organized training, analyzed barriers, developed EBP, applied evidence, and collected data. Three nursing research experts, all with master’s degrees and trained in evidence-based practices, were responsible for evidence retrieval, quality assessment, summarizing evidence, data analysis, and report writing.

#### Critical appraisal of evidence and audit criteria development

Three nursing researchers with expertise in evidence-based nursing independently performed critical appraisal of the included studies, using standardized critical appraisal tools: the AGREE II instrument for guidelines, AMSTAR 2 for systematic reviews, the Cochrane Risk of Bias 2.0 tool for the RCT, and JBI critical appraisal checklists for the expert consensus and clinical decision document. Disagreements were resolved through consensus discussions involving the corresponding author (a District Nurse Manager trained in evidence-based methodology) and an additional expert in evidence-based medicine. A total of 11 articles met the inclusion criteria: 1 clinical decision document [20], 4 guidelines [21–24], 1 expert consensus [25], 4 systematic reviews [26–29], 1 randomized controlled trial [30], The evidence-based project team systematically extracted relevant evidence content and sources, adhering to the principles of selecting high-grade, high-quality, and most recently published evidence. The extracted evidence covered five domains: organizational management, risk perception assessment, individualized intervention, training and education, and informatization and visualization, yielding 22 best practice recommendations. Of these, 18 recommendations were deemed suitable for clinical application following feasibility, appropriateness, meaningfulness, and effectiveness evaluation. Based on the best evidence and clinical scenario analysis, the team converted these 18 recommendations into 20 audit criteria, defining the audit targets and developing corresponding audit methods (Table 1).

**Table 1:** Best Practice recommendations and audit criteria

Best practice recommendations	Audit criteria	Audit target	Audit method
1. Identify relevant committees, organizations, or individuals to establish a formal fall - prevention support system and organizational framework. Utilize implementation science strategies to ensure the successful implementation, monitoring, and continuous improvement of the fall - prevention/injury - reduction program. 2. Organizational members should include: administrative personnel, service management staff, clinical healthcare providers, educators, personnel responsible for policy and quality improvement, and staff in charge of environmental maintenance and cleaning. Each professional should fulfill their respective responsibilities and promote fall prevention within the healthcare institution.	1. The hospital should establish a hospital-level falls safety management organizational structure. This structure should include at least the Medical Affairs Department, Nursing Department, Pharmacy Department, and Logistics Support Department.	Baseline review = 1 hospital Follow up = 1 hospital	Review the organizational structure documents of the hospital
	2. The department should establish a department - level organizational structure for fall safety management. This structure shall include, at a minimum, medical and nursing management personnel, clinical staff, and cleaning personnel, and corresponding job responsibilities shall be formulated.	Baseline review = 7 wards, Follow up = 7wards	Document review and interview method
3. Timing for Assessment: A fall risk perception assessment shall be performed upon the patient’s admission and repeated with any change in the patient’s condition. 4. Assessment Tools: The Fall Risk Perception Questionnaire for Patients (FRPQ) should typically be used for routine assessment, whereas for patients with spinal cord injury, the use of the Spinal Cord Injury-Falls Concern Scale (SCI-FCS) is recommended.	3. For inpatients identified as high - risk, nurses shall guide them to complete the Fall Risk Perception Questionnaire for Patients (FRPQ) for risk perception assessment upon admission and after any change in their condition.	Baseline review = 210 patients, follow-up audit = 210 patients	Review nursing records
5. Assessment tools should be integrated into the Electronic Medical Record (EMR) system, enabling nurses to conduct assessments efficiently.	4. Embedding fall risk perception assessment tools into electronic medical record (EMR) systems.	Baseline review = 7 wards Follow up = 7 wards	View the electronic medical record system
6. Healthcare providers proactively assess inpatients’ awareness and understanding of their fall risk level.	5. For high fall-risk patients, nurses should use positive language to communicate with them and routinely assess their awareness and understanding of their fall risk level.	Baseline review = 70 nurses Follow up = 70 nurses	Review nursing records and on-site observation and interview patients
7. A fall risk perception assessment should be incorporated into the multifactorial fall risk assessment for older adults. Healthcare providers should inquire about older adults’ perspectives on falls, the causes of falls, future risks, and how to prevent falls.	6. For inpatients assessed as high fall-risk, nurses should routinely assess: (1) the patient’s awareness of their own fall risk factors; (2) the patient’s understanding of relevant fall prevention measures; (3) the patient’s attitude and willingness to participate in fall prevention measures.	Baseline review = 210 patients, follow-up audit = 210 patients	Review nursing records and on-site observation And interview patients
8. Implement individualized multifactorial interventions and self-management support based on assessment of individual risk factors, risk perception, and preferences. 9. Collaborate with inpatients and their caregivers to evaluate the care plan and modify it as needed.	7. Based on the assessment of risk factors, risk perception, and personal preferences of high - risk inpatients, nurses should develop individualized, multifactorial fall prevention interventions with inpatients and their caregivers. They should also dynamically and timely adjust these interventions following events such as surgery or other changes in the patient’s condition.	Baseline review = 70 nurses Follow up = 70 nurses	on-site Observation Review nursing records and interview patients
	8. Nurses should implement individualized, multifactorial fall prevention interventions for high-risk hospitalized patients based on the plan.	Baseline review = 70 nurses Follow up = 70 nurses	on-site Observation Review nursing records and interview patients
10. Healthcare institutions should provide continuous education for all staff members (including but not limited to healthcare providers, nurses, nursing assistants, volunteers, dietitians, patient transport personnel, and caregivers).	9. Each unit should conduct fall prevention training for physicians, nurses, patient transport personnel, cleaning staff, and others at least once every six months.	Baseline review = 7 wards Follow up = 7 wards	review training records and interview staff (2 physicians, 2 nurses, 2 accompanying examiners, 2 nursing aides, 2 cleaning staff)
11. Train nursing staff to identify drugs that increase the risk of falls and enhance inpatients’ awareness of related risks.	10. Nurses have a knowledge rate of ≥ 85% regarding the names of high-risk medications for falls and related precautions used by patients.	Baseline review = 70 nurses Follow up = 70 nurses	Interview with nurses
12. Provide fall prevention education for all elderly individuals (≥ 65 years) and other high-risk populations.	11. Nurses provide targeted fall prevention education to patients and/or their families who are assessed as high-risk.	Baseline review = 70 nurses Follow up = 70 nurses	Interview with the patient/family member
13. Healthcare staff should inform hospitalized patients at risk of falls about their fall risk level, main risk factors, and targeted prevention measures.	12. Patients at high risk for falls and/or their family members are aware of their risk level.	Baseline review = 210 patients, follow-up audit = 210 patients	Interview with the patient/family member
	13. The awareness rate of risk factors among high-risk patients and/or their family members is ≥ 85%.	Baseline review = 210 patients, follow-up audit = 210 patients	Interview with the patient/family member
	14. The awareness rate of individualized fall prevention measures among high-risk patients and/or their family members is ≥ 85%.	Baseline review = 210 patients, follow-up audit = 210 patients	Interview with the patient/family member
14. Ensure that health education is delivered in a variety of formats (oral, written, and electronic, such as one - to - one or group health education, educational leaflets, handbooks, new media, or the internet) and in plain language.	15. Nurses provide fall prevention health education to high-risk patients using two or more methods (e.g., verbal instruction, video playback, distribution of brochures, etc.).	Baseline review = 70 nurses Follow up = 70 nurses	on-site observation
15. Embed fall prevention interventions within the Electronic Medical Record (EMR). 16. It is recommended to implement an evidence - based health education information system to deliver individualized fall prevention interventions to inpatients.	16. Fall prevention interventions are embedded in the Electronic Medical Record (EMR) system.	Baseline review = 7 wards Follow up = 7 wards	View the electronic medical record system
	17. The Electronic Medical Record (EMR) system can automatically generate and deliver personalized fall prevention measures based on a patient’s multifactorial fall risk assessment.	Baseline review = 7 wards Follow up = 7 wards	View the electronic medical record system
17. Place a clearly visible “Fall Risk Drug” warning label on the bedside area or medication container of any inpatient taking high - fall - risk medications to raise their awareness of the risk.	18. Attach a “High Fall-Risk Medication” warning sign to the drug packaging of any such medications used by inpatients during hospitalization.	Baseline review = 7 wards, Follow up audit = 7 wards	on-site observation
18. As part of the prevention strategy, consider implementing the use of colored identification bands, monitoring devices, assistive tools, and protective equipment for elderly inpatients identified as high fall-risk.	19. High fall-risk l patients should wear patient gowns, wristbands, or other items with “High Risk of Fall” warning labels.	Baseline review = 210 patients, Follow-up audit = 210 patients	on-site observation
	20. High fall-risk patients should have high-risk fall warning labels placed at the bedside.	Baseline review = 210 patients, Follow-up audit = 210 patients	on-site observation

#### Baseline audit

A baseline audit was conducted from June to July 2024, involving 210 patients at high risk of falling and 70 nurses across seven inpatient wards. The audit evaluated three main areas: compliance with best practice guidelines for fall risk awareness enhancement among high fall-risk inpatients, patient outcomes—including fall incidence rates, fall-related injury rates, Fall Risk Perception Questionnaire (FRPQ) scores, patient knowledge, attitude, and behavior scores and nursing staff knowledge and attitudes regarding fall risk awareness. The details were as follows.

### Process level

At this level, we assessed compliance with the 20 audit criteria spanning multiple domains of fall risk awareness enhancement. For clarity, audit criteria refer to specific, measurable standards of care derived from best practice recommendations; the checklist refers to the structured data collection instrument used to systematically record whether each criterion was met; and the audit refers to the systematic process of evaluating implementation against these predetermined standards. Audit team members used the self-developed “Inpatient Fall Risk Awareness Improvement Checklist” to conduct daily on-site observations, interviews with nurses and patients, and medical record reviews. Observable practices, documentation, and system performances that met the audit criteria were marked with “√” on the verification form, while non - compliance was marked with “×.” The compliance rate for each criterion was calculated using the following formula: Compliance rate = (Number of times the criterion was met / Total number of reviews) × 100%. Data collection methods included interviews, on - site verification, and review of the electronic medical record system.

### Patient level

At this level, we evaluated patient outcomes related to falls and fall risk awareness.

#### Fall incidence and injury severity

The research team prospectively documented all falls occurring during the hospitalization period, including fall frequency and severity. A fall was defined as an event resulting in a patient coming to rest inadvertently on the ground, floor, or other lower level [31]. Fall-related injuries were categorized as none, minor (requiring no or minimal intervention), moderate (requiring medical intervention), or severe (resulting in fractures, head trauma, or death) [32]. Data was collected through incident reports and the adverse event reporting system.

#### Fall risk perception

Patients at high risk of falls were assessed using the Chinese version of the Fall Risk Perception Questionnaire, which was translated and culturally adapted by domestic scholars [33]. This questionnaire consists of 26 items across three dimensions: environmental factors (13 items), personal - mobility factors (8 items), and personal - chronic condition factors (5 items). The Chinese version exhibited strong psychometric properties, with a scale - level content validity index (S - CVI) of 0.867, an average item - level content validity index (I - CVI) of 0.909, and a Cronbach’s alpha coefficient of 0.942. Patients typically required 5–10 min to complete the questionnaire. Research team members distributed the questionnaire to patients for self-completion. For patients who were unable to complete it independently, a team member read each item aloud and recorded the responses to ensure full comprehension. The questionnaire was administered once upon admission and again prior to discharge. 

#### Knowledge, attitude, and behavior regarding fall prevention.

Patients’ knowledge attitude and Behavior (KAB) regarding fall prevention were assessed using a 33-item questionnaire [34], which includes three dimensions: knowledge (11 items), attitude (9 items), and practice (13 items). The questionnaire demonstrated good reliability (Cronbach’s α > 0.8 for the overall scale) and validity (KMO = 0.933, Bartlett’s test *p* < 0.001). Trained research team members administered the questionnaire to patients for self-completion prior to discharge, with assistance provided as needed.

### Practitioner level

At this level, we assessed nurses’ knowledge and attitudes regarding fall risk awareness.

#### Nurses’ knowledge and attitudes regarding fall risk awareness

A self - designed test paper (Questionnaire 1: Questionnaire on the Awareness of Fall Risk among Nursing Staff) was used to assess the nurses’ mastery of evidence related to fall risk awareness. The detailed questionnaire is provided in Supplementary File 1. The assessment was carried out on 70 nurses. The test covered five key aspects with yes/no questions: the concept of fall risk awareness, understanding of fall risk awareness, familiarity with assessment tools, knowledge of how to conduct an assessment, and prior training received. Additionally, three items regarding attitudes toward fall risk awareness were included, using a five - point Likert scale.

### Phase 2: Design and implementation of strategies to improve practice

Based on the baseline audit findings, barriers to improving fall risk awareness were systematically identified and categorized into six domains using the JBI Getting Research into Practice (GRiP) framework. Through two rounds of expert consultation with a 20-member multidisciplinary Evidence Team, corresponding implementation strategies were developed, refined, and validated for feasibility and importance (both rated 5/5 on a 5-point Likert scale). The intervention design was grounded in the Theory of Planned Behavior [35], which posits that behavioral change is mediated through sequential modifications in knowledge, attitudes, and perceived behavioral control. The strategies were implemented over three months across five dimensions: organizational support, system optimization, staff training, patient education, and EMR-integrated warning systems. The barriers and corresponding implementation strategies are summarized in Table 2.

**Table 2:** barriers and corresponding implementation strategies

Barriers	Strategies	Resources	Outcomes
1. **Patient-**related: Limited awareness of fall risk; optimistic/fatalistic attitudes; cognitive/ literacy barriers; reluctance to seek help.	• Integrate FRPQ into the admission workflow as a structured conversation • Develop multi - format educational materials (pictogram leaflets, self - check cards, family guides, videos) • Mandate at least 2 education methods per patient with EMR documentation • Place bedside visual aids • Monitor awareness rates monthly to reach a target of at least 85%	• FRPQ-embedded EMR module • Education toolkit (leaflets, cards, video) • Standardized checklist • Bedside visual aids	Patients/families achieve ≥ 85% awareness of risk level, factors, and prevention measures; FRPQ completed within 24 h.
2. **Healthcare professional-related**: FRPQ perceived as paperwork burden; knowledge gaps (especially junior nurses); lack of interdisciplinary training; no standardized checklist or certification.	• Establish role - specific training every 6 months for all personnel. • Train nurses in FRPQ use and motivational interviewing. • Link effective engagement to reduced call bell use and falls. • Implement pre - and post - training assessments (≥ 85% medication knowledge target). • Introduce specialist certification for advanced competency.	• Training curriculum • Senior nurse mentors • Competency tools • Certification program	All relevant staff complete training every 6 months; nurse medication knowledge ≥ 85%; specialist certification established.
3. **Assessment tool and workflow**: FRPQ not integrated into EMR; no automatic generation of personalized measures; inconsistent documentation standards.	• Embed the FRPQ module into the EMR admission workflow. • Enable the automatic generation of personalized measures and push them to workstations and bedside screens. • Establish unified documentation standards across departments. • Monitor the completion rates and documentation quality monthly.	• IT specialist support • EMR enhancement • Standardized templates • Audit team	FRPQ fully integrated into EMR; personalized measures auto-generated; consistent documentation across departments.
4. **Educational and communication resources**: Lack of standardized, literacy-appropriate materials; insufficient instructors; over-reliance on verbal methods.	• Develop a multilingual, multi - literacy education toolkit • Train dedicated patient education specialists • Deploy videos on ward televisions and bedside screens • Mandate ≥ 2 methods per patient (verbal + video/brochure) • Monitor delivery and documentation via EMR audits	• Health literacy specialist • Multimedia resources • Ward TV/bedside screens • Printed materials	Standardized toolkit available; ≥2 methods used for 100% of high-risk patients; all education documented in EMR.
5. **Medication safety communication**: Inadequate labeling; no warnings on packaging; patient unaware of medication-related fall risk; nurse knowledge below 85%.	• Establish pharmacist - led medication review and discharge education. • Attach “High Fall - Risk Medication” warning labels to the packaging. • Integrate EMR alerts that prompt education during administration. • Verify patient understanding in medication reconciliation. • Conduct regular nurse knowledge assessments.	• Pharmacist time • Warning labels • Information cards • EMR alert system	100% high-risk medications labeled; all patients educated on medication-related fall risk; nurse knowledge ≥ 85%.
**6. Organizational and environmental**: No unified management structure; untrained third-party personnel; absent or inconsistent environmental warning cues.	• Establish hospital- and department-level multidisciplinary committees with patient representatives. • Develop a quality monitoring framework (process, outcome, awareness criteria). • Conduct monthly data review sessions. • Train cleaners/escorts with six - month refreshers. • Implement an environmental cue system (bedside screens, warning gowns/wristbands, posters, broadcasts).	• Hospital leadership • Committee members • Patient representative • Training materials for third-party staff • Warning labels/gowns/posters • Broadcast system	Unified management structures functional; 100% third-party personnel trained; environmental cue system fully implemented.

Note. FRPQ = Fall risk perception questionnaire; EMR = Electronic medical record

### Phase 3: Follow-up audit post-implementation of change strategy

Following the implementation of the evidence-based intervention bundle (derived from the 18 best practice recommendations identified through systematic critical appraisal of 11 studies in Phase 1, and operationalized via GRiP barrier analysis and expert validation in Phase 2), a second round of quality review, identical in content to the baseline audit, was conducted from October 2024 for three months. This review involved 70 nurses and 210 patients across seven units, all of whom were at high risk of falls and had fall risk awareness.

Data was entered and analyzed using SPSS version 26.0. Normally distributed measurement data were expressed as mean (SD), and comparisons between groups were performed using the independent samples t-test. Frequencies and percentages were used to describe count data. Comparisons of categorical variables between groups were performed using the chi-square test. Statistical significance was defined as *p* < 0.05.

## Results

**Fig. 1:**
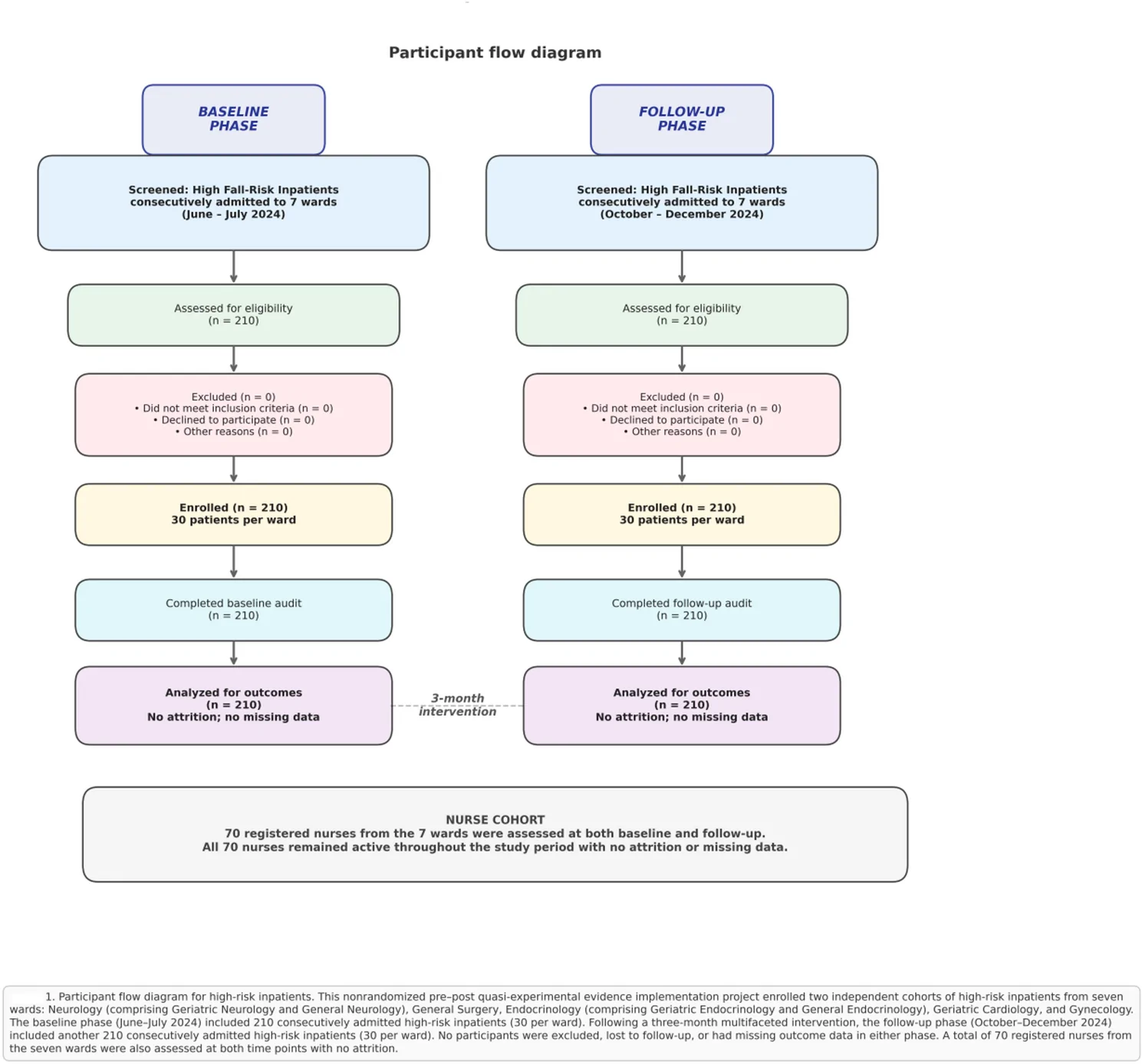
Participant flow diagram for high fall-risk inpatients. This nonrandomized pre–post quasi-experimental evidence implementation project enrolled two independent cohorts of high fall-risk inpatients from seven wards: Neurology (comprising Geriatric Neurology and General Neurology), General Surgery, Endocrinology (comprising Geriatric Endocrinology and General Endocrinology), Geriatric Cardiology, and Gynecology. The baseline phase (June–July 2024) included 210 consecutively admitted high fall-risk inpatients (30 per ward). Following a three-month multifaceted intervention, the follow-up phase (October–December 2024) included another 210 consecutively admitted high fall-risk inpatients (30 per ward). No participants were excluded, lost to follow-up, or had missing outcome data in either phase

A total of 420 high-risk inpatients were enrolled across the two phases: 210 in the baseline audit (June–July 2024) and 210 in the follow-up audit (October–December 2024), with 30 patients per ward in each phase. All enrolled patients completed the study, and no outcome data were missing in either phase (Fig. 1). Additionally, 70 registered nurses from the seven wards were included in both the baseline and follow-up assessments; all 70 nurses remained active throughout the study period with no attrition or missing data.

### Process level

#### Execution rates of different audit criteria before and after EBP

Following evidence-based practice implementation, 16 of the 20 audit criteria demonstrated significant improvement (*P* < 0.01). Criterion 1 showed no change from baseline, and criterion 2 and criterion 7 showed improvement that did not reach statistical significance. Notably, nine criteria achieved 100% compliance post-implementation, and seven criteria increased from 0% compliance at baseline to 100% compliance post-implementation. (Table 3).

**Table 3:** Execution rates of different audit criteria before and after EBP

Audit criteria	Before EBP(%)	After EBP(%)	X^2^	*p*
Criterion 1: Hospital-level fall safety management structure	100	100	0	1
Criterion 2: Department-level fall safety management structure	4 (57.14)	7 (100)	1.697	0.193
Criterion 3: FRPQ assessment upon admission and condition change	0 (0)	67 (95.71)	128.493	<0.001
Criterion 4: FRPQ embedded in electronic medical record system	0 (0)	7 (100.00)	10.296	<0.001
Criterion 5: Positive language and routine risk awareness assessment	0 (0)	59 (84.29)	101.975	<0.001
Criterion 6: Patient awareness of risk factors and prevention measures	50 (71.43)	69 (98.57)	20.224	<0.001
Criterion 7: Co-development of individualized multifactorial fall prevention plan with patient and caregiver	62 (88.57)	60 (85.71)	0.255	0.614
Criterion 8: Implementation of individualized multifactorial fall prevention interventions based on the plan	47 (67.14)	68 (97.14)	21.475	<0.001
Criterion 9: Fall prevention training every 6 months for all staff	0 (0)	7 (100.00)	10.296	<0.001
Criterion 10: Nurse knowledge of high-risk medications (≥ 85%)	48 (68.57)	69 (98.57)	22.943	<0.001
Criterion 11: Targeted fall prevention education for patients/families	47 (67.14)	68 (97.14)	21.475	<0.001
Criterion 12: Patient/family awareness of personal fall risk level	143 (68.10)	190 (90.48)	32.024	<0.001
Criterion 13: Patient/family awareness of specific fall risk factors (≥ 85%)	111 (52.86)	177 (84.29)	48.125	<0.001
Criterion 14: Patient/family awareness of prevention measures (≥ 85%)	107 (50.95)	164 (78.10)	33.794	<0.001
Criterion 15: Health education using ≥ 2 delivery methods	61 (87.14)	70 (100.00)	7.600	0.006
Criterion 16: Fall prevention interventions embedded in EMR	0 (0)	7 (100.00)	10.296	<0.001
Criterion 17: EMR auto-generation of personalized prevention measures	0 (0)	7 (100.00)	10.296	<0.001
Criterion 18: High fall-risk medication warning labels on inpatient drug packaging	0 (0)	7 (100.00)	10.296	<0.001
Criterion 19: High-risk patients wearing warning labels (gowns/wristbands)	0 (0)	7 (100.00)	10.296	<0.001
Criterion 20: Bedside high-risk fall warning labels	0 (0)	7 (100.00)	10.296	<0.001

Note. The full descriptions of Criteria 1–20 are provided in Table 1

### Patient level

#### The incidence rate of falls and the rate of fall-related injuries before and after EBP

Before EBP implementation, one fall occurred (incidence rate: 0.48% among the 210 baseline patients) resulting in one minor injury. After EBP implementation, no falls occurred among the 210 post-implementation patients, yielding a fall incidence rate of 0% and an injury rate of 0%.

#### Fall risk perception questionnaire scores before and after EBP

At both pre-implementation and post-implementation assessments, fall high-risk patien: ts demonstrated significantly elevated fall risk perception at discharge relative to admission (p < 0.01). Evidence implementation was associated with a significant increase in fall risk perception compared to the pre-implementation period (p < 0.01) (Table 4).

#### Knowledge, attitude, and behavior scores before and after EBP

After EBP implementation, fall high-risk patients scores in the three dimensions of fall prevention knowledge, attitudes, and behaviors were significantly higher than before EBP, with statistically significant differences (P < 0.01) (Table 5).

**Table 4:** Patient FRPQ scores before and after EBP

Period	Fall Risk Perception Score (at Admission) (*n* = 210)	Fall Risk Perception Score (at Discharge)(*n* = 210)
Before EBP	15.50 (12.93)	16.8 (12.69)
After EBP	20.21 (13.52)	28.15 (19.76)
*t*	-3.1	-6.3118
*p*	0.002	< 0.001

Note: Data are presented as mean (SD). FRPQ = Fall Risk Perception Questionnaire

**Table 5:** Patient knowledge, attitude, and behavior scores before and after EBP

Dimension	group	mean (SD)	t	*p*
Knowledge aspect	Before EBP	1.31 (0.75)	4.607	0.000**
	After EBP	1.63 (0.69)		
Attitude aspect	Before EBP	1.29 (0.75)	5.096	0.000**
	After EBP	1.64 (0.66)		
Behavioral aspects	Before EBP	1.28 (0.73)	4.904	0.000**
	After EBP	1.62 (0.66)		

Note: *p < 0.05, **p < 0.01, Data are presented as mean (SD). EBP = Evidence-based practice

### Practitioner level

#### Nurses’ knowledge regarding fall risk awareness before and after EBP

Results showed that the percentage of nurses answering “Yes” correctly to five fall risk awareness-related knowledge items was significantly higher after than before EBP implementation, with statistically significant differences (p < 0.01) (Table 6).

#### Nurses’ attitudes regarding fall risk awareness before and after EBP

The scores of nursing staff in three dimensions of fall risk awareness-related attitudes were significantly higher after intervention compared to before intervention, with statistically significant differences (P < 0.01) (Table 7).

#### Harms and unintended consequences

No adverse events or unintended negative consequences related to the intervention were observed during the study period. Specifically, no patient reported increased anxiety or fear of falling due to the risk awareness assessment or warning labels. Nurse feedback indicated that the additional documentation and assessment tasks were manageable within routine workflows, and no staff reported increased workload burden or burnout related to the EBP implementation.

**Table 6:** Nurses’ knowledge regarding fall risk awareness before and after EBP

knowledge	After EBP ( *n*%)		Before EBP ( *n*%)		*p*
	Yes	Deny	Yes	Deny	
knowledge 1	55(78.6)	15(21.5)	9(12.9)	61(87.1)	0.000**
knowledge 2	50(71.4)	20(28.6)	15(21.5)	55(78.6)	0.000**
knowledge 3	59(84.3)	11(15.7)	6(8.6)	64(91.4)	0.000**
knowledge 4	53(75.7)	17(24.3)	8(11.4)	62(88.6)	0.000**
knowledge 5	52(74.3)	18(25.7)	12(17.1)	58(82.9)	0.000**

Note *p < 0.05, **p < 0.01

**Table 7:** Nurses’ attitudes regarding fall risk awareness before and after EBP

dimension	Group	Mean (SD)	t	*p*
Perceived importance of fall risk awareness	Before EBP	2.03 (1.00)	6.078	0.000**
	After EBP	3.23 (1.32)		
Recognition of assessment value	Beforer EBP	2.01 (1.23)	4.162	0.000**
	Afte EBP	3.00 (1.55)		
Belief in education efficacy	Before EBP	1.71 (0.97)	6.138	0.000**
	After EBP	2.99 (1.44)		

Note. *p < 0.05, **p < 0.01

## Discussion

To our knowledge, this study represents one of the first attempts to systematically translate evidence on fall risk awareness into a structured, multi - dimensional clinical protocol specifically designed for high - risk inpatients and complements traditional fall prevention approaches. Unlike traditional fall prevention programs that primarily focus on environmental modifications and nursing surveillance, our intervention actively engages patients in understanding their personal fall risk through evidence - based strategies, thereby addressing a critical gap in patient - centered fall prevention.

The findings are consistent with significant improvements across process, patient, and practitioner level outcomes: 16 of 20 audit criteria showed significant improvement, patient FRPQ scores increased significantly at both admission and discharge, and nurse knowledge and attitudes improved substantially. The fall incidence rate was 0.48% (1/210) at baseline and 0% (0/210) post-implementation. Given the pre-post design and very low baseline fall rate, the reduction in fall incidence should be interpreted cautiously and does not support a strong causal claim. However, concurrent improvements in process compliance and patient awareness support the plausibility of a potential effect. These findings contribute to the growing body of evidence supporting patient-centered approaches to prevention in high-risk populations.

### EBP improves nurses’ execution rates for evidence-based criteria

Scholars are increasingly recognizing the importance of patients’ active awareness of fall risks during hospitalization, advocating for early cognitive intervention to enhance risk awareness [36, 37]. Although awareness has grown, standardized evidence-based guidelines detailing specific measures to improve fall risk awareness have been lacking. To address this gap, the nursing department-initiated evidence interpretation meetings to promote the EBP project and foster interdisciplinary collaboration among physicians, nurses, pharmacists, and logistics personnel. Leveraging leadership support, the team conducted comprehensive barrier analyses and developed targeted implementation strategies.

Following EBP implementation, 16 of the 20 audit criteria demonstrated significant improvement, with nine criteria achieving 100% compliance and seven criteria increasing from 0% to 100% compliance. These improvements demonstrate enhanced application of evidence in clinical practice and support the feasibility of the EBP approach. The substantial improvements in previously zero-compliance criteria are particularly noteworthy, suggesting that the barrier analysis and targeted strategies effectively addressed previously insurmountable implementation challenges. These findings provide valuable references for other institutions seeking to improve fall risk awareness among hospitalized patients.

### EBP projects improve patients’ fall risk awareness and their knowledge, attitudes, and behaviors regarding falls

EBP implementation significantly improved high-risk hospitalized patients’ awareness of fall risks and enhanced their knowledge, attitudes, and behaviors regarding fall prevention. Prior to implementation, patients’ Fall Risk Perception Scores were 15.50 (12.93) at admission and 16.80 (12.69) at discharge. Following the EBP intervention, these scores increased significantly to 20.21 (13.52) at admission (*t* = -3.10, *p* = 0.002) and 28.15 (19.76) at discharge (*t* = -6.31, *p* < 0.001). These findings indicate that standardized organizational training, multimodal teaching strategies, and informatization methods effectively enhance patients’ awareness of fall risk. This aligns with Haines et al. [38], who reported that multimodal education improved fall risk awareness in elderly patients, but extends their work by demonstrating the additional benefit of EMR-integrated personalized feedback and structured patient engagement protocols.

Furthermore, significant improvements were observed across all three dimensions of fall prevention: knowledge increased from 1.31 (0.75) to 1.63 (0.69) (*p* < 0.001), attitudes from 1.29 (0.75) to 1.64 (0.66) *(p* < 0.001), and behaviors from 1.28 (0.73) to 1.62 (0.66) (*p* < 0.001). This tripartite improvement aligns with the Theory of Planned Behavior, which posits that behavioral change is mediated through sequential modifications in knowledge, attitudes, and perceived behavioral control [39]. Individualized health education and diverse teaching modalities—including verbal instruction, visual aids, demonstration, and return demonstration—not only increased fall high-risk inpatients’ knowledge of fall risks but also positively transformed their attitudes, resulting in improved preventive behaviors.

The pronounced improvement in patients’ fall risk awareness from admission (*t* = -3.1) to discharge (*t* = -6.31) reflects a cumulative effect of iterative education during hospitalization, which is consistent with the findings of Price et al. [40] that spaced learning and repeated reinforcement are more valuable than single-session approaches in achieving sustained behavioral change.

### EBP projects improve nurses’ knowledge and attitudes regarding fall risk awareness

After EBP implementation, nurses demonstrated a significant increase in their awareness rates regarding the concept of fall risk awareness, assessment tools, and intervention methods (*p* < 0.01). This indicates that targeted training and multiple forms of teaching mode can effectively enhance the knowledge level of professionals, providing strong support for improving clinical nursing quality [25], and improves the nurses’ attention to the significance of fall risk awareness in fall prevention. Notably, the enhancement of nurses’ attitudes toward the significance of fall risk awareness appeared to follow their knowledge gains, consistent with the Theory of Planned Behavior positing that attitude change is predicated upon belief modification [41]. As nurses developed deeper understanding of risk factors and assessment protocols, their professional commitment to fall prevention strengthened accordingly. This knowledge-attitude synergy is critical because positive attitudes predict sustained behavioral engagement in patient education and proactive risk mitigation [42].

Consequently, improved nurse competency creates a cascade effect: when nurses possess sophisticated risk awareness capabilities, they communicate risks more effectively to patients, thereby altering patients’ awareness of fall vulnerability. This indicates that during evidence-based practice, standardized organizational training can effectively improve nurses’ knowledge of fall risk awareness in hospitalized patients, ultimately fostering a patient safety culture that extends beyond individual practitioners to benefit the entire care continuum.

## Conclusion

This study selected 420 inpatients at high risk of falls and 70 nurses from a tertiary hospital in Changsha. Through evidence-based methods, 22 pieces of best evidence were integrated, and a three-month intervention was implemented across five aspects: organization, systems, training, education, and informatization. The results showed that after the intervention, compliance rates for 16 audit criteria significantly improved, healthcare professionals’ related knowledge and beliefs increased, patients’ knowledge, attitudes, and practices regarding fall prevention improved. These findings provide preliminary support for integrating patient-centered risk communication into routine fall prevention programs for high-risk inpatients, although confirmatory evidence from rigorous randomized controlled trials remains necessary.

## Limitations and prospects

This study has several limitations. First, the nonrandomized pre–post quasi-experimental design with independent cohorts limits causal inference; without randomization or a concurrent control group, temporal confounders (e.g., seasonal variation, policy changes) cannot be excluded. Second, selection bias may exist because we prioritized departments with the highest fall incidence, including only the seven units with the highest rates over the preceding three years to maximize the potential impact of evidence application. Third, duo to the limited research period and research sampling in the evidence application process of this study, we did not conduct stratified analyses of intervention effects across different disease types or patient populations.

Future research should address these limitations by employing a randomized controlled trial or concurrent controlled design to strengthen causal inference, expanding the sample scope across more diverse departments and patient populations, and incorporating cognitive function assessment to further explore personalized intervention strategies for diverse populations.

## Supplementary Information

Below is the link to the electronic supplementary material.


Supplementary Material 1



Supplementary Material 2


## Data Availability

Data is provided within the manuscript or supplementary information files.
