# Supplementary material for: Enhancing fall risk awareness among high fall-risk inpatients: a best practice implementation project
Source: BMC Nurs. 2026 Jun 18;25:772. doi: 10.1186/s12912-026-04891-7 (PMC13528142; doi:10.1186/s12912-026-04891-7)
Supplement: Supplementary file 1 [file 12912_2026_4891_MOESM1_ESM.docx]

### ****Questionnaire 1: Nurses' Knowledge and Attitudes Regarding Fall Risk Awareness****

**Instructions:**

This questionnaire assesses your knowledge and attitudes toward fall risk awareness. It consists of two sections: Section A (Knowledge) with yes/no questions and Section B (Attitudes) with Likert scale items. Please answer all questions based on your current understanding and beliefs.

### ****Section A: Knowledge of Fall Risk Awareness****

*Please mark "Yes" or "No" for each item*

| **No**. | **Item** | **Yes** | **No** |
| --- | --- | --- | --- |
| 1 | Have you heard of the concept of fall risk awareness? | □ | □ |
| 2 | Do you understand what fall risk awareness means? | □ | □ |
| 3 | Are you familiar with assessment tools for measuring fall risk awareness? | □ | □ |
| 4 | Do you know how to conduct a fall risk awareness assessment with patients? | □ | □ |
| 5 | Have you received prior training specifically on fall risk awareness? | □ | □ |

### ****Section B: Attitudes Toward Fall Risk Awareness****

*Please circle that best represents your level of agreement*

| **No**. | **Statement** |  |  |  |  |  |
| --- | --- | --- | --- | --- | --- | --- |
| 1 | Fall risk awareness is highly important for preventing falls in hospitalized patients. | Strongly Disagree | Disagree | Neutral | Agree | Strongly Agree |
| 2 | Assessing fall risk awareness is a key strategy in fall prevention. \| | Strongly Disagree | Disagree | Neutral | Agree | Strongly Agree |
| 3 | Providing targeted health education based on patients' perceived fall risk awareness helps prevent falls. | Strongly Disagree | Disagree | Neutral | Agree | Strongly Agree |
