# Supplementary material for: Enhancing fall risk awareness among high fall-risk inpatients: a best practice implementation project
Source: BMC Nurs. 2026 Jun 18;25:772. doi: 10.1186/s12912-026-04891-7 (PMC13528142; doi:10.1186/s12912-026-04891-7)
Supplement: Supplementary file 2 [file 12912_2026_4891_MOESM2_ESM.docx]

****The Chinese version of Fall Risk Perception Questionnaire for Patients****

****Instructions:**** Please indicate whether each of the following statements is a risk factor for your fall based on your actual situation. Mark (√) in the box that applies to you.

****Department:**** ___________ ****Gender:**** ___________ ****Age:**** ___________

****Occupation:**** ___________ ****History of fall(s):**** □ Yes □ No

| Item | Strongly Disagree | Somewhat Disagree | Somewhat Agree | Strongly Agree |
| --- | --- | --- | --- | --- |
| I am at risk of falling because I have had experiences of falling in the past. | □ | □ | □ | □ |
| I am at risk of falling because I have general weakness. | □ | □ | □ | □ |
| I am at risk of falling because I lack strength in my legs. | □ | □ | □ | □ |
| I am at risk of falling because I have poor balance. | □ | □ | □ | □ |
| I am at risk of falling because I have poor vision. | □ | □ | □ | □ |
| I am at risk of falling because I have poor hearing. | □ | □ | □ | □ |
| I am at risk of falling because I feel dizzy. | □ | □ | □ | □ |
| I am at risk of falling because I do not get enough sleep. | □ | □ | □ | □ |
| I am at risk of falling because I have difficulty concentrating. | □ | □ | □ | □ |
| I am at risk of falling because I feel unsteady on my feet . | □ | □ | □ | □ |
| I am at risk of falling because I need to get up and go to the bathroom at night. | □ | □ | □ | □ |
| I am at risk of falling because I need need help when moving. | □ | □ | □ | □ |
| I am at risk of falling because I wear shoes that do not fit. | □ | □ | □ | □ |
| I am at risk of falling because I do not keep bed rails up. | □ | □ | □ | □ |
| I am at risk of falling because the bed is too high | □ | □ | □ | □ |
| I am at risk of falling because the surroundings are disorganized. | □ | □ | □ | □ |
| I am at risk of falling because I use a walking aids. | □ | □ | □ | □ |
| I am at risk of falling because I use medical devices, such as IV tubing and an IV pole. | □ | □ | □ | □ |
| I am at risk of falling because the lighting in patient rooms is very low. | □ | □ | □ | □ |
| I am at risk of falling because I take sedatives for tests and procedures. | □ | □ | □ | □ |
| I am at risk of falling because I am fasting for tests and procedures. | □ | □ | □ | □ |
| I am at risk of falling because the environment of the hospital is unfamiliar. | □ | □ | □ | □ |
| I am at risk of falling because there are thresholds in the hallway and bathroom. | □ | □ | □ | □ |
| I am at risk of falling because the bathroom or shower floor is wet. | □ | □ | □ | □ |
| I am at risk of falling because The floor of the hospital is slippery. | □ | □ | □ | □ |
| I am at risk of falling because there are not enough grab bars and handrails in the hospital. | □ | □ | □ | □ |

**Source and Authorization Note:** The Chinese culturally adapted version was translated and validated by Bao et al. (2022), and the present study has obtained authorization from the Chinese version developers to use this instrument. The original English version is available upon request from the original author Jeeeun Choi. See original publication: Choi J, Choi SM, Lee JS, et al. Development and validation of the fall risk perception questionnaire for patients in acute

care hospitals [J]. J Clin Nurs, 2021, 30(3-4):406-414. doi: 10.1111/jocn.15550. Epub 2020 Nov 19.
